# Training lip force by oral screens. Part 3: Outcome for patients with stroke and peripheral facial palsy

**DOI:** 10.1002/cre2.282

**Published:** 2020-04-17

**Authors:** Madeleine Wertsén, Manne Stenberg

**Affiliations:** ^1^ Hospital Dentistry, Special Dental Care Sahlgrenska University Hospital Mölndal Sweden; ^2^ Deparment of Signals and Systems Chalmers University of Technology (Retired) Gothenburg Sweden

## Abstract

The aim of this study was to investigate whether training with an oral screen can improve oral motor function in patients with stroke and peripheral palsy. The participants in the study were eight patients with orofacial dysfunction after stroke, included 7–14 months after onset, and seven patients with peripheral palsy, included 14–28 months after onset. A customized oral screen in acrylic was made for each participant. The screen had a tube around the handle to allow air to pass when measurements were made of the perioral muscle force. When measuring the ability to suck, the hole was sealed with wax. The participants trained with the oral screen two times daily for 5 min. Measurements were made at baseline, after 1 month and thereafter every third month until no further improvement was achieved. Measurements were made with two different instructions, to squeeze and to suck. In the stroke group, muscles around the mouth improved when pouting and smiling; these participants also achieved statistically significant changes when sucking. For the peripheral palsy group, little improvement could be seen when pouting and smiling. However, these patients reported less or no drooling, and the measurements for sucking increased significantly for six of the seven patients. The first recorded significant change was seen in the stroke group after 4 weeks training and in the group with peripheral palsy after 6 weeks. Training with a custom‐made oral screen can significantly improve perioral muscle force and the ability to create negative intraoral pressure. The patients reported less leakage in saliva, drink, and food as well as fewer bite injuries and less food accumulation.

## 1 INTRODUCTION

Orofacial dysfunction can be defined as affected facial expression, impaired intelligibility, eating and drinking problems, and drooling (Bakke, Bergendal, McAlister, Sjögreen, & Åsten, 2007). Patients who have impaired orofacial functions due to stroke, tumor surgery, Bell's palsy, or infections often suffer from problems such as leakage of saliva, beverage, and food due to reduced lip force (LF) (Hägg, Olgarsson, & Anniko, 2008). It has long been known that longstanding orofacial dysfunction can result in teeth moving out of their position and malocclusion due to lack in equilibrium between muscular activity in the lips and cheeks on the outside of the dental arch and the tongue on the inside (Tomes, 1873). It has also been shown that malocclusion is more frequent among children swallowing without tooth contact especially in combination with tongue thrust (Melsen, Attina, Santuari, & Attina, 1987). Reduced tongue control is common and leads to several difficulties such as poor oral clearance and bite wounds (Gabre, Norrman, & Birkhed, 2005; Veis & Logemann, 1985). Furthermore, patients may have articulation problems and difficulty transporting saliva and food through the oral cavity due to reduced tongue pressure against the hard palate (Hirota et al., 2010). Tongue function plays an important role in mastication by moving in synchrony with jaw movements as well as adjusting pressure against the hard palate (Hori, Ono, & Nokubi, 2006). Reduced swallowing capacity can lead to choking and aspiration (Veis & Logemann, 1985). Also, not being able to communicate or to have a meal with family and friends is a severe disability that affects the social life considerably. Several studies have shown the importance of being able to create negative pressure to effect proper swallowing (Engelke, Jung, & Knösel, 2011; Santander, Engelke, Olthoff, & Völter, 2013). In a study by Hirota, post‐stroke patients with dysphagia showed a general decline in tongue pressure during swallowing compared with non‐dysphagic patients (Hirota et al., 2010).

When studying rehabilitation of patients after stroke and peripheral palsy, it is important to wait until the spontaneous recovery has ceased to be able to determine if the treatment is successful. Following stroke, no further spontaneous improvement in activity of daily living can be expected even for the most severely affected patients after 6 months (Jörgensen, Nakayama, Raaschou, & Olsen, 1995).

In a study of 2,570 patients with peripheral facial nerve palsy, 71% regained normal function within 3 months, and after 6 months no further normal mimical function was obtained. In the group with incomplete recovery, 12% had slight sequelae, 13% had moderate sequelae, and 4% had severe sequelae (Peitersen, 2002).

Hägg et al. showed that training with an oral screen can increase swallowing capacity and LF in stroke patients (Hägg & Anniko, 2008). By measuring the LF when the patient squeezes the oral screen, a value of the perioral muscle force is obtained. The maximum LF is dependent of the screen area. If the area is calculated, LF can be expressed in an oral screen pressure (OSP) quantity as force per unit area. This is useful when comparing measurements from screens with different sizes. By measuring the force when the patient sucks the oral screen, it is possible to get an estimation of the patient's ability to create negative intraoral pressure. Mean value and standard deviation for single measurements vary considerably between individuals. Thus, all measurements should be analyzed individually (Wertsén & Stenberg, 2017a; Wertsén & Stenberg, 2017b).

## 2 AIM

The aim of this study was to investigate whether training with an oral screen can improve oral motor functions in patients with stroke and peripheral palsy.

## 3 MATERIALS AND METHODS

The Ethics Committee of the University of Gothenburg approved the study (Dnr 508‐00), and it was performed in accordance with the Declaration of Helsinki. All participants gave their informed consent.

### 3.1 Participants

Totally, 19 patients with orofacial dysfunction, 10 with stroke, and 9 with peripheral palsy were included in the study. They had all been referred to the oral motor team, consisting of a dentist and a speech and language pathologist, at the Special Dental Care Unit, Sahlgrenska University Hospital, Mölndal. Four participants were dropped out at the time of calculating the results, two persons with stroke and two with peripheral palsy. For two subjects, baseline measurements were missing, and the other two did not turn up at the last visit and thus failed to submit their oral screens for measurements.

The eight remaining patients with stroke, five men and three women aged 23–84 years, were admitted to the study 7–14 months after onset, and one patient started training 22 years after onset. Seven patients had central facial palsy, four had persisting weakness on the left side and three on the right side, and one had suffered a brainstem hemorrhage resulting in a left‐side weakness. Seven patients had peripheral facial palsy, one man and six women aged 27–59 years, while three joined the study after having Bell's paralysis, one after a borrelia infection and two after surgery for acusticus neurinoma. One patient had undergone an operation because of a lymphoma in the brainstem. They were included 14–28 months after onset of the illness.

### 3.2 Inclusion criteria

- Patients were included a minimum of 6 months after onset of the illness.
- Patients had to be able to understand information and instructions.
- Patients had to be able to exercise with an oral screen regularly, by themselves or with assistance.

#### 3.2.1 Oral screen

A customized oral screen was made for each participant from the plaster model produced after impressions of the upper and the lower jaw at the first visit. The oral screen covered the oral vestibule from the distal surface of tooth 15 to the distal surface of tooth 25.

**Figure 1:**
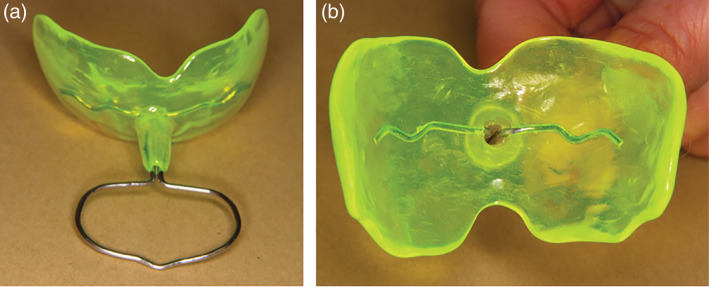
(a) Customized oral screen with a tube around the handle. (b) A hole through the tube allowed air to pass when measuring the force of the perioral muscles. The hole was sealed with wax when exercising

The screen was made of acrylic and had a handle of metal wire surrounded by a small tube in acrylic. At the base of the wire, there was a hole through the screen and the tube (Figure 1a,b).

This design allowed air to pass when measurements were made of the perioral muscle force. When exercising and measuring the ability to suck, the hole was sealed with wax.

### 3.3 Questionnaire

Before the visit, the patients filled out a questionnaire to explain their orofacial problems. The questions were about accidental biting, leakage, drooling, oral clearance, articulation, and swallowing. The answers were marked on a VAS scale ranging from “no problems” to “extreme problems.” Furthermore, the patients could, in their own words, describe how the oral motor dysfunction affected their social interactions.

### 3.4 Procedure

At the first visit, the oral motor function of tongue, jaw, and mimic muscles was assessed by the dentist and the speech and language pathologist, using a protocol with a four‐grade scale modified from the grading system by Peitersen that has five grades (Table 1). The modification done was to eliminate grade 2, “Slight—Only visible when patient grimaces,” as this grade does not lead to any noteworthy disability (Peitersen, 2002).

**Table 1:** Grades of dysfunction modified from the grading system by Peitersen, 2002

Grade	Degree of palsy	Description of palsy
0	None	Normal function
1	Moderate	Visible with small facial movements
2	Severe	Function just visible
3	Complete	No function

On the second visit, the patient was given the custom‐made screen. The patient and/or the assistant was instructed how to perform the exercise with the oral screen in the mouth of the patient. The instructions were to

1. Place the oral screen in the space between the teeth and the lips,
2. Grab the handle and pull the screen straight out while sucking as hard as possible,
3. Stop pulling when the screen is almost pulled out of the mouth,
4. Repeat steps 1 to 3 for 5 min twice a day.

A written instruction with pictures was given and explained. When the patient was familiar with the screen, the baseline measurements were made. After 1 month, the patients had a third appointment to check that the instructions were being followed. Thereafter, the patients visited the clinic every third month. Measurements were made at each visit, and changes of the originally reported symptoms were noted. At the last visit, the function was again assessed according to the protocol with the four‐grade scale (Table 1). The oral screen was kept in the clinic to calculate the area of the screen. When this had been done, the oral screen was returned to the patient.

#### 3.4.1 Lip force measuring

The LF meter LF 100 was used at every measurement. It is an electronic instrument measuring the maximum LF in Newtons over a set period of 10 s. A wire is connected to the oral screen and to a force transducer based on strain gauge registering forces from 0 to 250 N with a resolution of 1 N (0.4%) (Hägg et al., 2008; Hägg & Anniko, 2008; Wertsén & Stenberg, 2017a; Wertsén & Stenberg, 2017b). The patient was sitting in an upright position in a dental chair with arms and footrests and with the head against a headrest. The measuring procedure and the instructions were explained. Then the measurements were performed with and without suction. The instruction without suction was, “Hold the oral screen in your mouth and squeeze your lips as firmly as you can, while I pull it out.” The instruction with suction was, “Hold the oral screen in your mouth and suck it as hard as you can, while I pull it out.”

The participant placed the oral screen in the vestibulum. The wire was stretched in a straight angle perpendicular to an imaginary line between the nose and the chin of the participant, and an assistant signaled the start of the measuring period of 10 s. The examiner pulled the wire and gradually increased the power until the oral screen was pulled loose. The maximum value was noted. Measurements were made at baseline and then at every visit after 1 month and thereafter every third month until no further improvement was achieved. At every visit, the LF was measured three times in a sequence with the screen tube open and likewise three times with the screen tube closed with wax. All the values were used in the statistical analyses. Results from a group of healthy adults were achieved from a previous study by Wertsén & Stenberg (2017b) and were used to compare the results from this study.

#### 3.4.2 Procedure for measuring projected area of the oral screen

To be able to calculate the OSP, the area of the screen was determined by a projecting method (Wertsén & Stenberg, 2017a; Wertsén & Stenberg, 2017b). To calculate the size of the screen, every screen was placed on a piece of paper. From a perpendicular direction, the parallel projected contour could be identified and drawn on the paper, where a reference area with known size also was inserted. The result was scanned and explored in an image manipulation program.

### 3.5 Patient experience of treatment

Depending on the problems reported in the questionnaire, the patients were asked about the effect of the training with the oral screen. The answers were noted in the medical record.

### 3.6 Statistical analyses

The dataset was analyzed in MS Excel with StatPlus Analysis Toolpak (AnalystSoft). The OSP values were obtained by dividing force values by appropriate individual screen areas. Both the mean value at a certain occasion and an estimated standard deviation within the same occasion, also known as standard error of measurement (SEM), were calculated from a one‐way ANOVA analysis with the different times as the different groups. Original data from “Squeeze” measurements with open screens (*P*_*Sq*_) were treated separately from “Suck” measurements with closed screens (*P*_*Su*_). The additional OSP from suction (*P*_*Su+*_) was evaluated as the difference between the two mean values at every occasion.

(1) $$P_{\mathrm{Su}+}=P_{\mathrm{Su}}-P_{\mathrm{Sq}}.$$

The SEM values for the “Squeeze” measurements SEM_Sq_ were calculated as the square root of the mean square within groups (MSWG_Sq_) from the ANOVA analysis.

(2) $${\mathrm{SEM}}_{\mathrm{Sq}}=\sqrt{{\text{MSWG}}_{\mathrm{Sq}}}.$$

The SEM values for *P*_*Su+*_ were calculated as the square root of the sum of mean squares within groups for both the *P*_*Su*_ and the *P*_*Sq*_ measurements.

(3) $${\mathrm{SEM}}_{\mathrm{Su}+}=\sqrt{{\text{MSWG}}_{\mathrm{Sq}}+{\text{MSWG}}_{\mathrm{Su}}}.$$

In our case, we calculated the mean at every occasion from *m* = 3 measurements. With *k* = 3 occasions, we got in total at least *n = mk* = 9 measurements for each individual parameter. The smallest real difference (SRD) between two means at 95% confidence level could then be calculated as (ref. Part 14):

(4) $${\mathrm{SRD}}_{\text{mean}}=t_{.975,\mathrm{df}}∙\frac{\mathrm{SEM}}{\sqrt{m}}∙\sqrt{2},$$

where *t*_.975, *df*_ is the value of the t statistic with cumulative probability 0.975, and *df* = *n* − *k* degrees of freedom. In our case, we got *t*_.975, 6_ = 2.45 and

(5) $${\mathrm{SRD}}_{\text{mean}}=2.45∙\frac{\mathrm{SEM}}{\sqrt{3}}∙\sqrt{2}=2.0∙\mathrm{SEM}.$$

Since the number of occasions differed between the different patients, the actual value of *df* was considered when calculating the different SRD values.

## 4 RESULTS

### 4.1 Stroke group

In the stroke group, oral motor dysfunction affected the mimic muscles, mainly the muscles around the mouth, and for some patients the tongue was affected. At baseline, some patients had difficulties with coordination when changing rapidly and rhythmically between pouting and smiling. This improved considerably after training. Improvement assessed by the oral motor function protocol could be seen (Table 2). The main symptoms reported by the stroke patients were leakage of drink, drooling, accidental biting, and food retention. These symptoms improved the most after training, whereas articulation problems persisted. Five patients reported swallowing problems. Two reported that there were no problems after training and two that choking was less frequent, but that food consistency still had to be modified (Table 3). Several participants also described changes in their social lives.

**Table 2:** Oral motor function in patients with stroke assessed after protocol with four‐grade scale modified from the grading system by Peitersen, 2002(Table 1)

Patient	Tongue mobility		Pout		Smile		Elevation larynx	
	Start	End	Start	End	Start	End	Start	End
S1	3	1	1	0	2	0	0	0
S2	0	0	1	0	3	2	0	0
S3	3	2	3	1	2	2	0	0
S4	0	0	1	0	2	1	1	0
S5	0	0	1	0	2	1	0	0
S6	1	0	1	0	2	1	0	0
S7	0	0	0	0	1	0	0	0
S8	0	0	3	3	2	0	0	0

**Table 3:** Symptoms reported by patients with stroke before and after training

Patient	Accidental biting		Leakage		Drooling		Retention of food		Articulation		Swallowing	
	Start	End	Start	End	Start	End	Start	End	Start	End	Start	End
S1	1	0	1	0	1	0	1	0	1	1	0	0
S2	1	0	1	0	1	0	1	1	1	1	1	Better
S3	0	0	1	Less	1	Less	0	0	1	Better	1	1
S4	0	0	1	0	1	0	1	0	0	0	1	0
S5	1	0	1	0	1	0	1	0	Aphasia		0	0
S6	0	0	1	0	1	0	1	0	1	1	1	0
S7	0	0	1	0	0	0	1	0	1	Better	1	Better
S8	0	0	0	0	1	0	1	0	Aphasia		0	0

*Note:* 0 = No problem and 1 = Yes, has problem.

**Figure 2:**
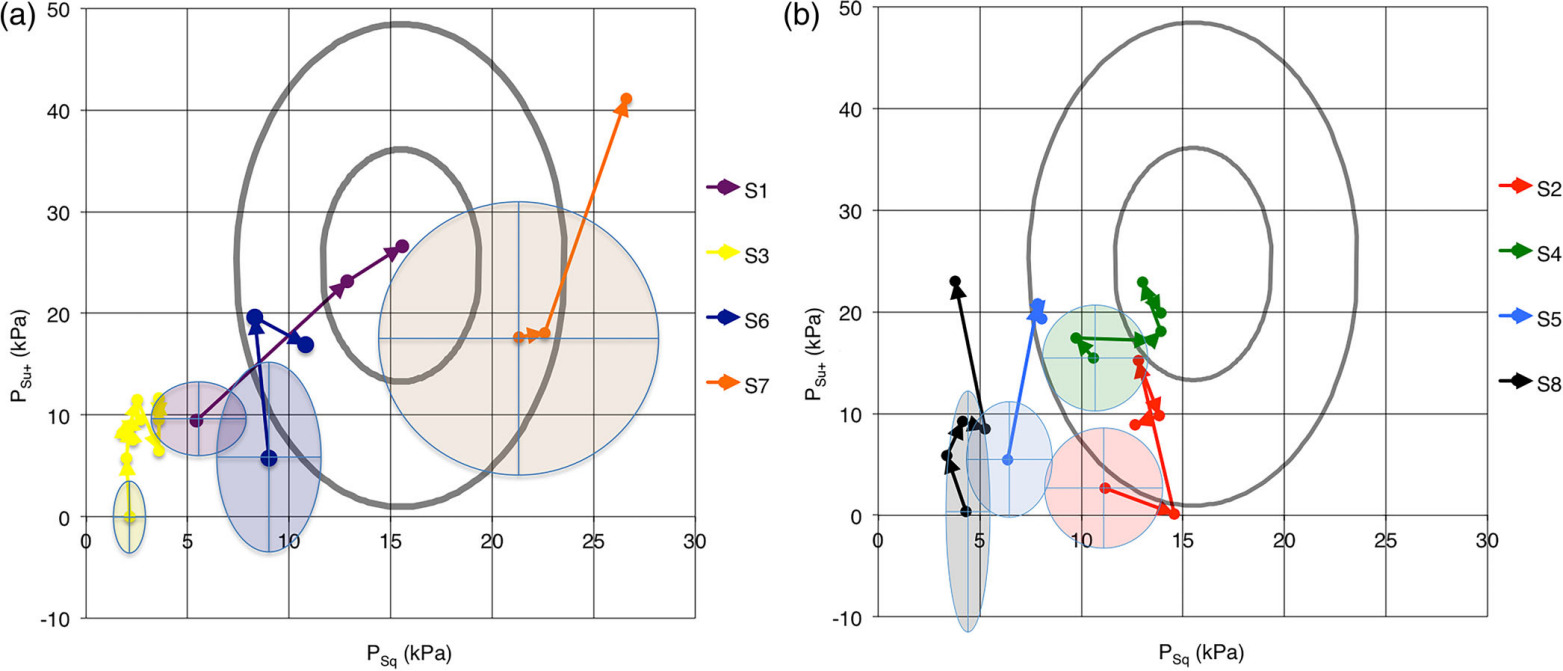
(a) Oral screen pressure changes as the result of training for different patients with stroke. Around every start value is an ellipse showing the area of smallest real difference (SRD) to indicate a statistically significant change. Inner ellipses show results from a healthy normal group (Wertsén & Stenberg, 2017a). The innermost ellipse corresponds to mean values 50% confidence ellipse, and the outer ellipse corresponds to 95% confidence ellipse. Patients S1, S3, S6 and S7. (b) Oral screen pressure changes as the result of training for different patients with stroke. Around every start value is an ellipse showing the area of SRD to indicate a statistically significant change. Inner ellipses show results from a healthy normal group (Wertsén & Stenberg, 2017a). The innermost ellipse corresponds to mean values 50% confidence ellipse, and the outer ellipse corresponds to 95% confidence ellipse. Patients S2, S4, S5 and S8

The result of training on the different OSP parameters can be seen in Table 4a,b and Figure 2a,b. The start values were lower than 95% of a group of healthy adults for six of the eight patients in the stroke group (Wertsén & Stenberg, 2017b). The variability at a certain occasion can be analyzed from the SEM values (Table 4a,b); for the *P*_*Sq*_ parameter, these varied as much as 0.5–3.5 kPa among the different patients, and for the *P*_*su+*_ parameter, the SEM values varied between 1.9 and 6.4 kPa. This implies that the SRD values differed among the patients, and Figure 2a,b show that criteria for significant change could vary both between patients and also with the two parameters for the OSP (*P*_*Sq*_ and *P*_*su+*_). The time dependance of recorded significant changes is shown in Table 5. Statistically significant improvements could be seen for all patients. The improvements were mainly in the *P*_*su+*_ parameter, but three patients (S1, S3, and S4) also showed significant improvement in the last recording of the *P*_*Sq*_ parameter. The first recorded significant changes could in many cases be seen after 4 weeks' training. For one patient, the first recorded significant change was seen after a comparatively long time, up to 52 weeks.

**Table 4:** Oral screen pressure data (*P*_*Sq*_ and *P*_*su+*_) for patients with stroke based on lip force measurements

a) Oral screen pressure *P*_*Sq*_ based on lip force measurements with the instruction, “Squeeze as hard as you can.”						
Patient	*P*_*Sq*_ start (kPa)	*P*_*Sq*_ end (kPa)	SEM (kPa)	*df*	SRD (kPa)	Change/SRD
S1	5.4	15.6	1.3	14	2.3	4.3
S2	11.2	12.6	1.6	10	2.9	0.5
S3	2.2	3.6	0.5	22	0.8	1.8
S4	10.6	13.9	1.5	12	2.6	1.3
S5	6.4	8.1	1.1	6	2.1	0.8
S6	9.0	10.8	1.3	6	2.6	0.7
S7	21.3	26.6	3.5	6	7.0	0.8
S8	4.3	3.8	0.6	10	1.1	−0.5

b) The additional oral screen pressure from suction *P*_*su+*_ evaluated as the difference between *P*_*su*_ (oral screen pressure “Suck”) and *P*_*sq*_ (oral screen pressure “Squeeze”).						
Patient	*P*_*Su*+_ start (kPa)	*P*_*Su*+_ end (kPa)	SEM (kPa)	*df*	SRD (kPa)	Change/SRD
S1	9.4	26.6	1.9	6	3.7	4.6
S2	2.7	8.9	3.3	10	5.9	1.1
S3	0.0	9.5	2.1	22	3.5	2.7
S4	15.5	19.9	2.9	12	5.2	0.8
S5	5.4	19.3	2.8	6	5.6	2.5
S6	5.8	16.9	4.7	6	9.3	1.2
S7	17.7	41.2	5.9	4	13.4	1.8
S8	0.4	23.0	6.4	10	11.7	1.9

*Note*: Standard error of measurement (SEM) is based on ANOVA analysis of all measurements. The smallest real difference (SRD) is calculated from the SEM value and t statistics with corresponding degree of freedom (*df*). Statistically significant changes are present for Change/SRD values greater than one.

**Table 5:** Time dependence of recorded statistically significant changes for patients with stroke

Patient	*P*_*Sq*_ change first recorded	Max *P*_*Sq*_ change recorded	Last *P*_*Sq*_ change recorded	*P*_*Su*+_ change first recorded	Max *P*_*Su*+_ change recorded	Last *P*_*Su*+_ change recorded
S1	7.4/4	11.1/20	10.1/104	13.8/4	17.2/104	17.2/104
S2	3.4/15	3.4/15	–/55	12.6/26	12.6/26	6.3/55
S3	1.4/172	1.4/172	1.4/240	5.7/15	11.6/208	9.5/240
S4	2.9/18	3.3/38	3.3/56	7.5/50	7.5/50	–/56
S5	–	–	–	15.4/10	15.4/10	13.9/29
S6	–	–	–	13.9/4	13.9/4	11.2/20
S7	–	–	–	23.5/12	23.5/12	23.5/12
S8	–	–	–	22.6/52	22.6/52	22.6/52

*Note*: Values are change/time. Change in kPa and time in weeks.

### 4.2 Peripheral palsy group

The main symptoms reported by the peripheral palsy group were leakage of drink and drooling. Three patients also reported difficulty swallowing. Oral motor dysfunction affected only the mimic muscles and especially the muscles around the mouth. Little improvement assessed by the oral motor function protocol could be seen (Table 6). However, the patients reported better function (Table 7). The measurements for sucking the oral screen increased significantly for six of the seven patients (Table 9).

**Table 6:** Oral motor function in patients with peripheral facial palsy assessed after protocol with four‐grade scale modified from the grading system by Peitersen, 2002(Table 1)

Patient	Tongue mobility		Pout		Smile		Elevation larynx	
	Start	End	Start	End	Start	End	Start	End
PFP1	0	0	1	1	1	1	0	0
PFP2	0	0	2	2	2	2	0	0
PFP3	0	0	2	1	1	1	0	0
PFP4	0	0	2	1	2	1	0	0
PFP5	0	0	3	2	2	1	0	0
PFP6	0	0	3	3	2	2	0	0
PFP7	0	0	3	3	3	3	0	0

**Table 7:** Symptoms reported by patients with peripheral palsy before and after training

Patient	Accidental biting		Leakage		Drooling		Retention of food		Articulation		Swallowing	
	Start	End	Start	End	Start	End	Start	End	Start	End	Start	End
PFP1	0	0	0	0	0	0	0	0	1	0	0	0
PFP2	1	0	1	0	1	0	1	0	0	0	1	0
PFP3	0	0	1	1	1	0	1	0	0	0	0	0
PFP4	0	0	1	1	1	0	0	0	0	0	0	0
PFP5	0	0	1	0	0	0	0	0	1	0	1	0
PFP6	0	0	1	0	1	0	1	0	0	0	0	0
PFP7	0	0	1	0	1	0	1	0	1	0	1	0

*Note*: 0 = No problem and 1 = Yes, has problem.

**Figure 3:**
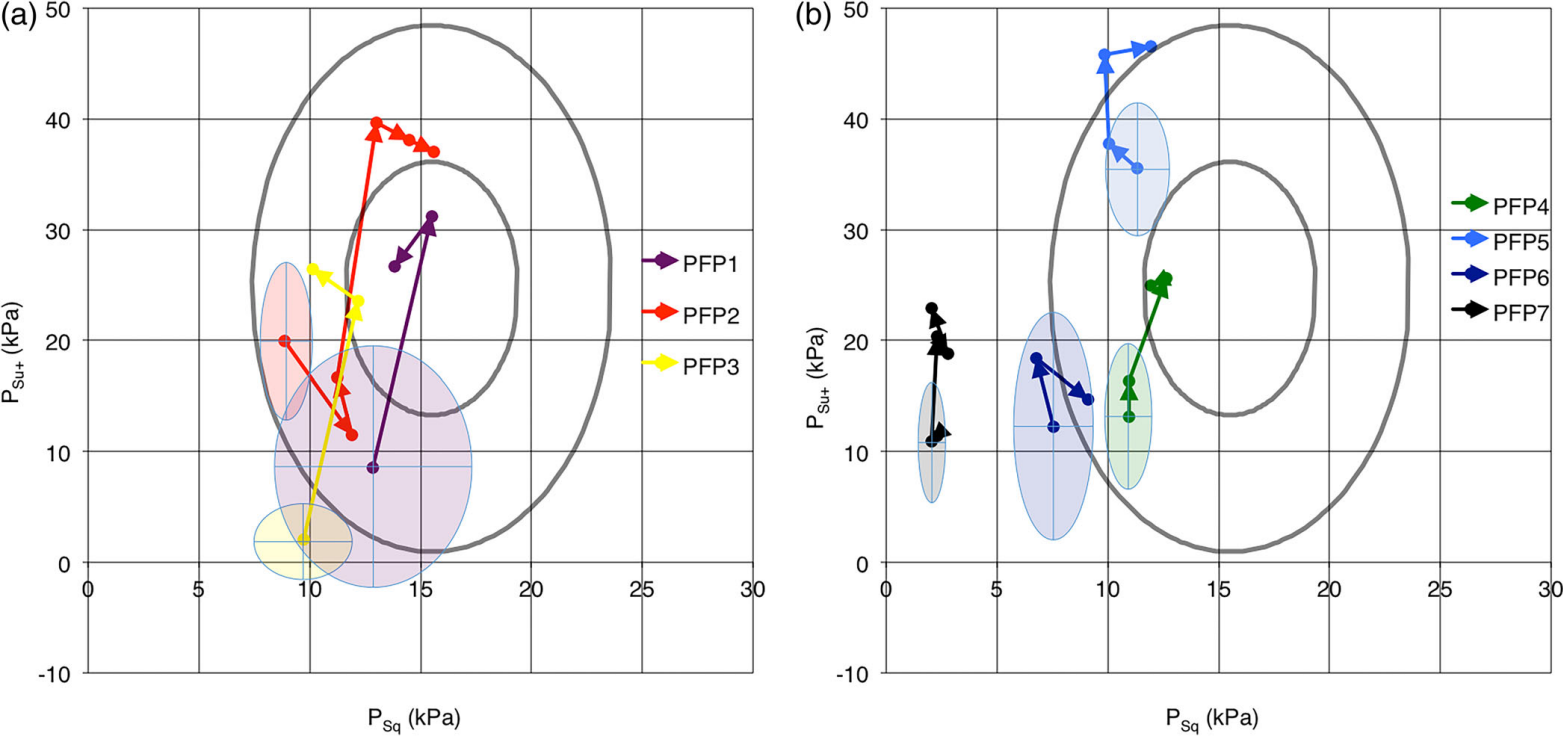
(a) Oral screen pressure changes as the result of training for patients with peripheral facial palsy. Around every start value is an ellipse showing the area of smallest real difference (SRD) to indicate a statistically significant change. Inner ellipses show results from healthy adults (Wertsén & Stenberg, 2017a). The innermost ellipse corresponds to mean values 50% confidence ellipse, and the outer ellipse corresponds to 95% confidence ellipse. Patients PFP1, PFP2 and PFP3. (b) Oral screen pressure changes as the result of training for patients with peripheral facial palsy. Around every start value is an ellipse showing the area of SRD to indicate a statistically significant change. Inner ellipses show results from healthy adults (Wertsén & Stenberg, 2017a). The innermost ellipse corresponds to mean values 50% confidence ellipse, and the outer ellipse corresponds to 95% confidence ellipse. Patients PFP4, PFP5, PFP6 and PFP7

For the peripheral palsy group, the result of training on the different OSP parameters can be seen in Tables 8 and 9 and Figure 3a,b. The start values were lower than 95% of the control group for three of the seven patients in the peripheral palsy group. The SEM value differed among patients with 0.7–1.5 kPa for the *P*_*Sq*_ parameter and 1.7–5.5 kPa for the *P*_*su+*_ parameter. As with the stroke group, statistically significant changes were mainly seen in the *P*_*su+*_ parameter. Six of seven patients showed significant improvements in the *P*_*Su*_ + parameter, but only three of seven patients showed significant improvements in the *P*_*Sq*_ parameter. The first recorded significant improvements were seen after 6 weeks' training. As with the stroke group, the first recorded significant changes could be seen after a comparatively long time, up to 44 weeks (Table 10).

**Table 8:** Oral screen pressure data *P*_*Sq*_ for patients with peripheral facial palsy based on lip force masurements

Patient	*P*_*Sq*_ start (kPa)	*P*_*Sq*_ end (kPa)	SEM (kPa)	*df*	SRD (kPa)	Change/SRD
PFP1	12.8	13.8	1.5	6	2.9	0.3
PFP2	8.9	15.6	0.7	6	1.3	5.0
PFP3	9.7	10.1	1.1	6	2.2	0.2
PFP4	10.9	12.0	0.6	8	1.1	1.0
PFP5	11.3	11.9	0.8	8	1.5	0.4
PFP6	7.6	9.1	1.0	6	2.0	0.8
PFP7	2.3	2.0	0.3	10	0.6	−0.4

**Table 9:** The additional oral screen pressure data from suction *P*_*su+*_ for patients with peripheral facial palsy based on lip force masurements

Patient	*P*_*Su*+_ start (kPa)	*P*_*Su*+_ end (kPa)	SEM (kPa)	*df*	SRD (kPa)	Change/SRD
PFP1	8.5	26.7	5.5	6	10.9	1.7
PFP2	19.9	37.0	3.6	6	7.1	2.4
PFP3	2.1	26.4	1.7	6	3.4	7.1
PFP4	13.1	24.9	3.5	8	6.6	1.8
PFP5	35.5	46.5	2.7	4	6.0	1.8
PFP6	12.2	14.7	5.1	6	10.3	0.2
PFP7	11.5	22.9	3.0	10	5.5	2.1

**Table 10:** Time dependence of recorded statistically significant changes for patients with peripheral facial palsy

Patient	*P*_*Sq*_ change first recorded	Max *P*_*Sq*_ change recorded	Last *P*_*Sq*_ change recorded	*P*_*Su*+_ change first recorded	Max *P*_*Su*+_ change recorded	Last *P*_*Su*+_ change recorded
PFP1	–	–	–	22.7/7	22.7/7	18.2/25
PFP2	3.0/6	6.7/79	6.7/79	19.7/44	19.7/44	17.1/79
PFP3	2.4/6	2.4/6	–/19	21.5/6	24.3/19	24.3/19
PFP4	1.7/18	1.7/18	–/42	12.5/18	12.5/18	11.8/42
PFP5	–	–	–	10.3/20	11.0/29	11.0/29
PFP6	–	–	–	–	–	–
PFP7	–	–	–	8.9/16	11.5/40	11.5/40

*Note*: Values are change/time. Change is in kPa and time is in weeks.

## 5 DISCUSSION

This study shows that training with a custom made oral screen can significantly improve perioral muscle force and the ability to create negative intraoral pressure. Also, the participants reported less leakage of saliva, drink, and food as well as fewer events with accidental biting and better oral clearance. The results were achieved even though the time in which improvement is considered possible had passed. These findings are consistent with the result of other studies (Hägg et al., 2008; Hägg & Anniko, 2008).

#### 5.1.1. Stroke group

At the start, all patients had problems with leakage of saliva and/or drink and food. At the end of the test period, all but one reported no problems of this kind. Six of eight participants started with poor measurements lying outside the outer ellipse corresponding to 95% confidence ellipse for participants with normal oral motor function (Figure 2). At the end of the training period, three of these had achieved a level within the 95% confidence ellipse and one even in the 50% confidence ellipse. This indicates that patients with oral motor dysfunction can benefit from training with an oral screen and that it is not necessary to reach measurements comparable to those of a normal healthy population to achieve improved oral motor function. Also, patients with severe oral impairment and patients with long‐standing oral motor dysfunction can improve their function. For patients with severe stroke, the training may have to continue for several years before any result can be achieved. Therefore, it is important to encourage the patients to carry on with their daily exercises.

#### 5.1.2. Peripheral palsy group

Estimated time for spontaneous recovery is 3–9 months. In this study, no patients were included until 1 year after the onset of the disease had passed. The heterogeneous cause of facial palsy has been a problem in other studies as well as in this study (Peitersen, 2002).

According to the results of the assessment of the oral motor function, the participants in the peripheral palsy group showed difficulties only in pouting and smiling. All but one had severe or complete degree of palsy on the affected side. While some in the stroke group had problems with their tongue mobility and elevation of the larynx, these problems were not observed in the peripheral palsy group.

Although there was not so much improvement in the assessed exterior oral motor function (pout and smile), the symptoms did improve. This is probably due to the improved ability to suck, as all patients in the group except one (PFP6) increased their force in sucking significantly. In this group, three participants were outside the 95% confidence ellipse at the start of the study. Only one remained outside, starting with very low measurements, but still reported improvement of the original symptoms.

### 5.2 Orofacial function

For some patients, the assessment of orofacial function after exercise shows that there still is a dysfunction, despite the patient reporting improved function. A possible explanation is that the patients had become accustomed to the dysfunction and learned to use their capacity so that the disability causes as little obstacle as possible. On the other hand, there could have been a true improvement in function, but the methods available to measure changes are too poor. Other common symptoms, such as accidental biting, poor oral clearance, and leakage of drink and food, are hard to test and must be determined by a structured interview. In this study, we did not test swallowing problems, as we mainly were interested in the oral motor dysfunction and the effect of training. All patients improved their suction force significantly, but the effect on the perioral force was less obvious. However, the perioral muscles are important in the process of swallowing, being a stable anterior lock in the space created to form negative pressure. Thus, measurement of the force when squeezing gives valuable information. Suction force is connected to the ability to create negative pressure, which is necessary to swallow (Engelke et al., 2011). A recent study showed that training with an oral screen can improve swallowing dysfunction among older people in intermediate care (Hägglund, Fält, Hägg, Wester, & Levring, 2019). A reduction in swallowing problems reduces the risk of choking and coughing during meals, which is an embarrassing situation when eating and limits social life. It also minimizes the risk of aspiration and developing pneumonia.

The duration of the study was up to 1 year, except for one participant who had suffered from a very severe stroke (S3), in whom the orofacial function was largely nonexistent at the start. For this patient, the training went on for 4 years. It was interesting to observe that even this very severe dysfunction could improve. To achieve this, an extremely motivated patient is required. In this study, the greatest improvement occurred within 13 weeks of exercise. This indicates that a training period of 4–8 months may be reasonable. However, individual patients with severe dysfunction may experience improvement after a very long training period.

### 5.3 Exercise instruction

In this study, the instruction to the participants was to exercise 5 min twice a day by sucking the screen as hard as possible and trying to pull it out of the mouth. In another study, the instruction was to squeeze 5–10 s with gradually increased pulling. The exercise was to be performed three times per session and three times a day (Hägg & Anniko, 2008). Despite the different schemes for training, improvements were obtained. It would be interesting to study what training method and training duration would be the most effective to achieve improvement.

It is also likely that patients, after long periods of training, get tired of exercising twice a day. This study has not followed the long‐term effect of training with an oral screen, but it shows that some of the patients were able to maintain the achieved LP while some lost some of their strength. Further studies are required to evaluate whether the achieved result remains when training ends, or if patients should be advised to continue to exercise a couple of times a week.

### 5.4 Limitations

Some possible weaknesses in this study are to be mentioned. First, to avoid spontaneous recovery, which could have affected the outcome of the study, no participant in the stroke group was included in the study until the estimated time for recovery had passed (Jörgensen et al., 1995). It would have been desirable to have a control group, but as shown in a previous study, the normal population varies considerably (Wertsén & Stenberg, 2017a). Thus, it was considered too difficult to find matched controls, and the results were instead correlated with data from healthy subjects in a previous study (Wertsén & Stenberg, 2017b). Second, the stroke patients in this study varied considerably in severity of their illness, as the area of injury had different positions and distribution in the brain. To describe the group in a better way, the patients could have been assessed according to the Barthel Index or the functional independence measure (FIM™). This might have given a clearer picture of the overall disability of the participants. However, despite the lack of grading of the stroke patients, all patients but one in this study improved their ability to create negative oral pressure. It is possible that patients with mild, moderate, or severe stroke would achieve different degrees of improvement. This would be an interesting area to investigate in a future study.

Third, the evaluation of the mimical function could have been performed with a well‐known and validated grading system like the Sunnybrook Facial Grading Scale (Baricich, Cabrio, Paggio, Cisari, & Aluffi, 2012). This system measures symmetry at rest, voluntary facial movements, and synkinesis (Fattah et al., 2015). Nevertheless, in this study we focused on the oral motor dysfunction and found it useful to use the grading system for peripheral palsy developed by Peitersen (Hägg & Anniko, 2008). For use in the daily clinical setting, it is important to find scales that are easy to use but also provide valuable information. We found that the modified scale by Peitersen is one.

## 6 SUGGESTIONS FOR FUTURE STUDIES

To find out how patients with different degrees of stroke and peripheral palsy would respond to training of oral motor dysfunction would give valuable information concerning the level of rehabilitation possible to achieve. An interesting field to investigate is the long‐lasting effect of oral motor training and if there might be a need for continues supportive training. As there are several different methods for training, comparing these would give valuable information to find the most efficient technique. This study has focused on stroke and peripheral palsy. Now it is important to go on with other groups with nonprogressive neurological disease and oral motor dysfunction.

## 7 CONCLUSION

For patients suffering from stroke, training with a custom‐made oral screen can significantly improve perioral muscle force and the ability to create negative intraoral pressure. It is not necessary to reach measurements comparable to those of a normal population to achieve improved function. The patients reported less leakage in saliva, drink, and food as well as fewer bite injuries and better oral clearance.

For patients with peripheral palsy, training with a custom‐made oral screen can significantly improve perioral muscle force and the ability to create negative intraoral pressure. Although, there was little improvement in the exterior oral motor function, such as pout and smile, the symptoms drooling and leakage improved.
